# Assessing morphological, developmental, and genetic responses of Hydropsychid caddisflies to Cry1Ab exposure

**DOI:** 10.1093/ee/nvag025

**Published:** 2026-04-07

**Authors:** Ethan P Bull, Scott P Egan, Jennifer L Tank, Elise D Snyder, Yufei Qi, Amanda Potts, Maryam Rajabi Faghihi, Pedro F P Brandão-Dias

**Affiliations:** Department of BioSciences, Rice University, Houston, Texas 77005, USA; Department of BioSciences, Rice University, Houston, Texas 77005, USA; Department of Biological Sciences, University of Notre Dame, Notre Dame, Indiana 46556, USA; Department of Biological Sciences, University of Notre Dame, Notre Dame, Indiana 46556, USA; Prairie Research Institute, Illinois Natural History Survey, Champaign, Illinois 61820, USA; Department of BioSciences, Rice University, Houston, Texas 77005, USA; Department of BioSciences, Rice University, Houston, Texas 77005, USA; Department of BioSciences, Rice University, Houston, Texas 77005, USA; Department of BioSciences, Rice University, Houston, Texas 77005, USA; School of Marine and Environmental Affairs, University of Washington, Seattle, Washington 98105, USA

**Keywords:** Trichoptera, ecotoxicology, GMO, nontarget, Bt

## Abstract

Transgenic crops expressing Cry proteins are widely cultivated to reduce crop damage from insect pests. However, Cry proteins can leach from transgenic crop detritus into nearby aquatic ecosystems, potentially affecting nontarget organisms. Here, we assess the impacts of exposure to a commonly used Cry protein, Cry1Ab, on net-spinning caddisflies (Hydropsychidae), a common and ecologically important group of stream insects. We collected and genetically barcoded 1,862 larval caddisflies from 25 streams across the Midwestern United States, comparing populations from Cry1Ab-positive and Cry1Ab-negative streams. We evaluated potential impacts associated with body size, developmental stage, species assemblages, and mitochondrial haplotype diversity. Overall, we observed limited effects of Cry1Ab exposure, including most comparisons showing no difference between Cry1Ab-positive and Cry1Ab-negative streams with the exception of second instar *Hydropsyche betteni*, which showed significantly reduced body size at Cry1Ab-positive sites. Mean developmental stage within species and species assemblages showed no consistent association with Cry1Ab. In contrast, environmental variables such as water temperature, stream width, and watershed land use were stronger predictors of body size, instar progression, and species assemblages. Haplotype networks revealed strong geographic structure but no genetic patterns related to Cry1Ab exposure. These results indicate that Cry1Ab exposure is not a major driver of morphological, developmental, or genetic variation in Hydropsychid caddisflies under natural field conditions. Environmental gradients remain the dominant structuring force, although further work is needed to assess potential context-dependent or cumulative effects of transgenic crop byproducts in aquatic ecosystems.

## Introduction

Over 90% of maize, cotton, and soybean fields in the United States (US) are planted with genetically engineered (transgenic) varieties (Dodson 2025). Globally, adoption of transgenic crops is similarly widespread, accounting for about 74% of soybean hectares, 79% of cotton, and 32% of maize production worldwide (ISAAA 2019). These crops can provide agricultural benefits by decreasing pest damage and the need for chemical pesticides (Fitt 2008, Smyth 2020). Most US maize is genetically engineered to express insecticidal proteins known as Cry toxins (Dodson 2025), a diverse family comprising multiple isoforms with distinct names and target specificities, such as Cry1Ab, Cry1Ac, Cry1F, Cry2Ab, and Cry3Bb1. Many Cry toxins target key agricultural pests within the insect order Lepidoptera (eg butterflies and moths), including the European corn borer (*Ostrinia nubilalis* [Hübner, 1796]), while others target coleopteran pests such as the corn rootworm (*Diabrotica virgifera* LeConte, 1868). These Cry toxins originate from *Bacillus thuringiensis* (Bt), a bacterium first isolated from Mediterranean flour moths (Martin and Travers 1989, Alba-Tercedor and Vilchez 2023), and form lethal oligomers in the midgut of susceptible insects, causing internal rupture and death (Ibrahim et al. 2010). They are generally considered highly effective because they specifically target pest species that directly ingest the crops while posing no known adverse effects on human health and reduced impact on nontarget insects like pollinators (Mendelsohn et al. 2003, Ibrahim et al. 2010).

In addition to its molecular specificity, Cry toxins have been previously shown to rapidly degrade in natural terrestrial and aquatic systems (Head et al. 2002, Clark et al. 2005, Griffiths et al. 2017, Brandão-Dias et al. 2021). This rapid degradation presents another major ecological advantage of transgenic maize compared to chemical pesticides that persist longer in the environment (Turusov et al. 2002, Goulson 2013). Despite the potentially rapid decay rates of Cry proteins, they are considered “*pseudo-persistent*” due to a constant resupply of the toxin that leaches from crop material (Griffiths et al. 2017). The release of Cry toxin over time via maize detritus and exudates leads to consistently detectable concentrations in streams, which may result in unintended consequences for aquatic organisms (Rosi-Marshall et al. 2007, Tank et al. 2010, Venter and Bøhn, 2016). Accordingly, several studies have investigated the effect of Cry toxins on nontarget invertebrates, with mixed results (Arias-Martín et al. 2016).

Caddisflies (order: Trichoptera) are of particular interest because they are a diverse group of aquatic insects with a wide range of ecological roles (Wiggins 1996) and given their close evolutionary relationship with Lepidoptera—the intended target of Cry toxins—they may be more vulnerable than other organisms to unintended adverse effects. The caddisfly life cycle consists of an obligate aquatic larval stage, and larvae of many species can be found in streams near fields containing Bt maize (Holzenthal and Calor 2017); thus, they are likely exposed to the toxin in these systems (Rosi-Marshall et al. 2007, Tank et al. 2010). Accordingly, some studies have experimentally evaluated the effect of Cry toxins on these insects. Rosi-Marshall et al. (2007) looked at the scraping caddisfly, *Helicopsyche borealis*, so named for its feeding behavior of scraping algae off rocks for food, and found increased mortality when individuals were experimentally exposed to Bt maize in lab settings. Rosi-Marshall et al. also observed that a shredding caddisfly, *Lepidostoma liba*, suffered sublethal fitness consequences through reduced growth rather than increased mortality. Conversely, Jensen et al. (2010) investigated the impact of Cry1Ab on the shredding caddisfly *Pycnopsyche scabripennis* and caddisflies in the *Lepidostoma genus*, but found neither significant difference in mortality nor adverse effects on growth. Finally, Pott et al. (2020) found that feeding shredding caddisflies in the genus *Chaetopteryx* and *Sericostoma* a diet spiked with Cry1Ab resulted in sublethal adverse effects to chronic Bt exposure. Thus, the effects of Cry toxins on caddisflies are likely taxon-dependent and may not be broadly generalized.

Despite multiple studies on diverse groups of caddisflies, the impact of Cry toxins on one of the most abundant subgroups, the net-spinners (Family Hydropsychidae, 159 species in North America; Hogan et al. 2025) remains unknown, and few have evaluated the impact of the toxin in nontarget organisms in their natural environment. Hydropsychids spin nets to capture particulate detritus as a food resource in streams, which can potentially include material from Bt maize such as pollen and leaf particles (Wiggins 1996). Due to their close proximity to agricultural plots containing Bt maize, it is possible that Hydropsychids ingest Bt maize material that falls into the stream, being thus potentially exposed to the active form of the toxin. In addition, given the consistent detection of Cry proteins in the water itself across these landscapes (Brandão-Dias et al. 2022), the animals could be exposed and affected by the toxins in their dissolved form (Kimura et al. 2023).

In this study, we examined whether Cry toxins from transgenic crops, specifically the variant Cry1Ab, are influencing natural populations of filtering Hydropsychid caddisflies. We sampled larval caddisflies across a broad geographic range in the Midwestern United States, comparing populations from streams with detectable Cry1Ab toxin to those where the toxin was absent. Our analysis considered multiple biological levels: individual traits (body size and developmental stage), community-level differences (species composition), and intraspecific genetic structure (mitochondrial haplotypes). We hypothesized that most variation would be driven by environmental factors such as water temperature, latitude, and stream characteristics, which may also influence insect growth and life cycle progression. However, if Cry toxins exert sublethal or selective effects on Hydropsychids, we expected to detect deviations from these environmental patterns in Cry-positive streams. Because different species can differ in physiology, feeding ecology, and toxin susceptibility, we further hypothesized that any Cry1Ab effects may be species-specific rather than uniformly expressed across the community. By integrating ecological and genetic data across multiple sites, our goal was to assess whether Bt-associated toxins contribute to measurable biological changes in Hydropsychid caddisfly populations *in situ*, beyond the effects of natural environmental variation.

## Materials and Methods

### Field Collection

**Fig. 1.**
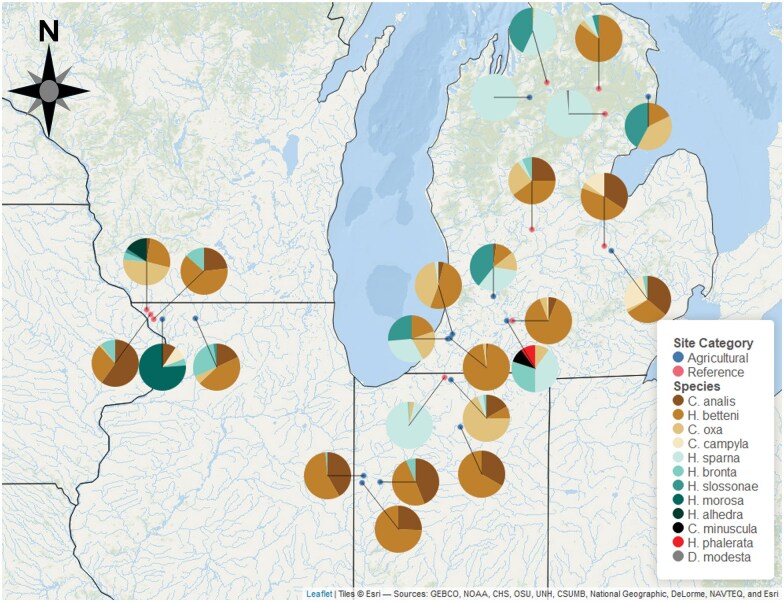
Collection sites colored by land-use of surrounding area. Shown in blue are the Nonagricultural collection sites and in pink are the Agricultural collection sites. Pie charts showing species composition at each site. Site coordinates are found in Table S2.

Hydropsychidae caddisflies were collected at 25 stream sites (Fig. 1, Table S2) across Illinois, Michigan, and Indiana in late July 2022. We chose these sites based on accessibility, category, and the presence of historical collections of Hydropsychid caddisflies from local museums. This was done for greater chance of collection success and for the possibility of future comparison of community composition. The sites included both agricultural and nonagricultural land use in the surrounding watershed; however, the identity of nearby crops (whether transgenic or not) was not known. Caddisflies were manually sampled by active search of suitable microhabitats within streams and were immediately placed in in vials of 100% ethanol upon collection. After collection was complete, we replaced the ethanol in the vials and placed specimens on ice for up to 12 h before storing them at −20 °C for later analysis. For each site, 2 collectors sampled for 45 min to 1 h, or until 100 insects were collected.

We also collected water samples for Cry1Ab protein analysis (see details below), nutrient quantification, and water column turbidity measurements. Turbidity was determined using an HQ2100Q portable turbidity meter (Hach), while water temperature and pH were recorded with a handheld pH meter (Oakton pHTestr 50). Following water sampling, stream velocity was measured at 5 points using a Marsh-McBirney Flo-Mate 2000 flow meter, along with manual measurement of stream width.

### Land Cover Determination

We quantified land cover at each sampling site and within their respective upstream watersheds using ArcGIS Pro v3.2 (Esri), following the workflow detailed in Fig. S1. We obtained land cover data for the year 2021 derived from Sentinel-2 satellite imagery, accessed via the ArcGIS Living Atlas of the World. The dataset represents classified land cover at 10-m spatial resolution and was used to identify the extent of urban and agricultural land upstream of each site. A circular buffer zone with a 6-km radius was created around each sampling location using the Buffer tool. To identify upstream watershed areas for each site, we utilized the USGS lidar-derived Digital Elevation Model (DEM). Specifically, we processed the DEM using the following ArcGIS hydrology tools in sequence: Fill (to remove elevation sinks), Flow Direction (to determine flow paths), Flow Accumulation (to inform minor adjustments of coordinates position), and Watershed (to delineate the upstream drainage area associated with each sampling site). Finally, we intersected the 6-km buffer zones with the delineated watershed polygons using the Intersect tool to define the precise area from which we extracted land cover data upstream of each sampling site.

### Cry1Ab Quantification in the Water

To assess the presence of Cry1Ab toxins in stream water, we collected three 45 ml water samples from each site, one from each edge of the channel, and one from the main flow in the center. We filtered each water sample using 0.45-µm PCTE filters, and stored water samples on ice for up to 12 h while in transport to the lab. Water samples were then stored at −20 °C until further analysis.

Cry1Ab concentrations from filtered water samples were quantified following the method described and validated by Strain et al. (2014). First, we concentrated water samples 30-fold using 30-kDa Amicon ultrafilters (EMD Millipore, Germany) by centrifuging twice at 2,000 × *g* for 30 min at 4 °C. The concentrated samples were then adjusted to 1,000 µl using phosphate-buffered saline with Tween buffer (Agdia Inc., Elkhart, Indiana) and quantified in triplicate using the Cry1Ab/1Ac enzyme-linked immunosorbent assay (ELISA) kit (Agdia Inc., Elkhart, Indiana). Each ELISA plate included a linear 8-point standard curve ranging from 0.12 to 16 ng/ml, prepared with purified Cry1Ab (MyBioSource Inc., San Diego, California). Plates were read at 650 and 450 nm using an Infinite 200 PRO plate reader (Tecan, Switzerland), with a 95% limit of detection (LOD) at 4.2 ng/l (Brandão-Dias et al. 2021). Water samples with concentrations below the LOD were treated as zeros in the final analysis. The Cry1Ab concentration for each site was calculated as the average concentration quantified across the 3 water samples collected per site.

### Morphological Measurements

To quantify individual traits (eg body size and developmental stage), we took images of side profiles of each individual caddisfly using a Leica M125 Stereoscope using Leica Application Suite (Fig. S3) paired with a Leica LED5000 Transmitted Light Base. We then measured insect body size using ImageJ (Schneider et al. 2012; Fig. S4). Additionally, we also measured insect head capsule width on each individual (Fig. S3), which was used to determine caddisfly instar for comparison (Panzavolta 2007). All insects were manipulated using sterilized tweezers that were cleaned between each individual with 5% bleach, water, and 70% ethanol to avoid cross-contamination of genetic material.

### Instar Determination and Instar Score

We determined instars using graphical analysis of head capsule length grouped by species (Holzenthal and Ríos-Touma 2012, Miess et al. 2022). Using head capsule width, we approximate what instar each caddisfly was in based on natural clusters in the data, seen in histograms (Fig. S5). These groups were then numbered starting with the smallest group of observed individuals getting assigned instar 1. The subsequent size comparisons among stream sites were then only made within individuals of the same instar and species.

To measure the development of our caddisflies across field sites, we calculated an “instar score” of each species for each site. This is defined as the average instar of individuals (sum(instars)/total number of individuals). These instar score values were compared across agricultural sites and site Bt content using a Mann–Whitney *U* test to compare means. We also performed an ordinary least squares regression on instar scores and water temperature.

### Comparison of Morphological Fitness

A student’s *t*-test was conducted to determine statistical differences in body area within each caddisfly instar and species. We compared with multiple, species-specific tests because without separating by species, we could not determine if differences in body size were due to the effector (Bt presence or site type) or species-specific ontogeny. Assumptions of normality were assessed using a Shapiro–Wilk test for normality. We looked at the differences between individuals found in reference streams (ie sites with little to no human agricultural land use around them; Fig. 1) and agricultural streams (ie sites with moderate to heavy agricultural land use; Fig. 1). We also compared individuals from streams containing measurable Cry1Ab concentrations to and sites that did not contain Cry1Ab. Significance values were adjusted using the Bonferroni correction to account for multiple testing. All statistical analyses were conducted using R 4.1 (R Core Team 2016) and performed at a significance level of α = 0.05. The data were visualized using the ggplot2 package (Wickham et al. 2025).

In addition to the species- and instar-specific tests described above, we modeled body area as a function of Cry1Ab and ancillary environmental predictors using a Gaussian generalized linear mixed model (GLMM) to account for nonindependence among observations. The GLMM was fit by forward selection of explanatory variables using the lme4 and MuMIn packages in R (Bartoń 2025, Bates et al. 2025). Certain arcGIS traits were dropped if their correlation with another trait exceeded 0.7 (Figs S9 and S10). The model formula was generated from the *dredge* function and was as follows:


Body area ∼ Cry1Ab+Lat+pH+Temp+Turb+Crops+Built+Width+Vel+Depth+(1 | species_instar),

where the response variable was the body area of the caddisflies, and fixed effects included, respectively, the water column Cry1Ab concentration, site latitude, water pH, water temperature, water turbidity, percent of crops in the watershed (arcGIS), and the percentage of impervious surface in the surrounding watershed (arcGIS), stream width, average water velocity of the stream, and the average stream depth. Random variables were the instar of the specimen and the species*. Dredge* used AIC to determine the best fitting model.

### Comparison of Species Compositions

We compared species compositions of caddisfly communities at each site using Non-Metric Multidimensional Scaling (NMDS), and used the Bray–Curtis dissimilarity matrix to assess differences in species composition among the individual streams. The analysis was conducted using the *metaMDS* function in the *vegan* package in R (Oksanen et al. 2025). We also compared various drivers influencing caddisfly community composition among streams for the collection sites on species composition using permutational multivariate analysis of variance (PERMANOVA) with the *adonis2* function in the R package *vegan*.

### Species and Haplotype Determination

We extracted DNA from each individual caddisfly semi-destructively using the 96-DNeasy Blood and tissue protocol (QIAGEN), following the manufacturer protocols. During specimen manipulations for morphological analyses, we removed a leg from each insect to facilitate permeation of Proteinase-k and lysis buffer into whole insects and facilitate DNA extraction. The exoskeleton and tissues remaining after digestion were then preserved in ethanol 90% at −20 °C as vouchers.

For species identification, we genetically barcoded each individual by amplifying and sequencing the mitochondrial COI (Cytochrome Oxidase I) gene. For amplification, reactions consisted of 12.5 µl of Apex 2X RED Taq Master Mix, 1.5 mM MgCl2 (Genesee Scientific), 1.0 µl of LCO1490/HCO2198 primer mix (Folmer et al. 1994) at 10 µM, 0.25 µl of extracted DNA, and molecular grade water for a final reaction volume of 25 µl. The reactions began with a denaturation step lasting 2 min at 95 °C, followed by 35 cycles of denaturation for 30 s at 94 °C, annealing at 52 °C for 15 s, and extension at 72 °C for 45 s. After these cycles, there was a final extension step lasting 2 min at 72 °C, followed by a hold at 4.0 °C. We then verified the positive amplification of each individual using a 1.5% agarose gel. The resulting amplicons were subjected to Sanger sequencing at the University of Arizona Genetics Core. Subsequently, the sequences were manually edited and aligned using MEGA 11 software (Tamura et al. 2021), and species identification was performed by BLAST against sequences in NCBI’s nr database. We considered our generated sequences to belong to the same species as reference sequences when their genetic distance was less than 2%, and we followed the species nomenclature from the Checklist of Nearctic Trichoptera (Rasmussen and Morse 2023).

To further assess population structure, we analyzed intraspecific haplotype variation using the aligned COI barcode sequences. Specifically, we examined how haplotype distributions related to sampling geography and the presence or absence of Cry1Ab. Haplotype networks were constructed using the TCS algorithm (Clement et al. 2002) as implemented in POPART (Leigh and Bryant 2015), allowing visualization of genealogical relationships among individuals within each species.

## Results

### Total Collections and Species Identified

We collected 2,138 Hydropsychidae caddisfly larvae across 25 stream sites, of which 1,862 individuals were successfully barcoded and identified to species. A complete list of records by site is provided in the Supplementary Material in Darwin Core format, and all generated sequences are deposited on GenBank under accession numbers PX427848 to PX429571.

We documented 12 different species including (from most frequent to least) 770 *Hydropsyche betteni* Ross 1938; 335 *Hydropsyche sparna* Ross 1938; 289 *Cheumatopsyche analis* (Banks 1903); 191 *Cheumatopsyche oxa* Ross 1938; 145 *Hydropsyche slossonae* Banks, 1905; 67 *Hydropsyche bronta* Ross 1938; 40 *Cheumatopsyche campyla* Ross 1938; 16 *Hydropsyche morosa* Hagen 1861; 6 *Hydropsyche alhedra* Ross 1939; 1 Cheumatopsyche minuscula (Banks, 1907); 1 *Diplectrona modesta* Banks 1908; and 1 *Hydropsyche phalerata* Hagen 1861 (Fig. S8).

Of the 12 observed species, 6 (*H. betteni*, *H. sparna*, *C. analis*, *C. oxa*, *H. slossonae*, and *H. bronta*) were collected frequently enough in both reference and impacted sites, so that instars could be assigned based on head capsule width (Table 1).

**Table 1.** Individuals per species per instar for the 6 species where instars could be assigned based on head capsule width distribution (Fig. S5, Table S1)

Species	Instar	Count	Species	Instar	Count
***Cheumatopsyche analis*** 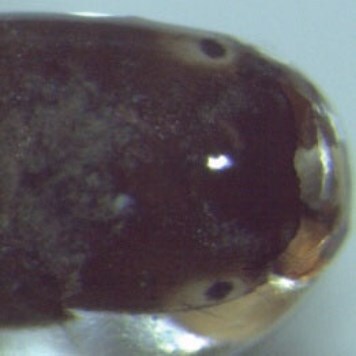	1	9	*Hydropsyche betteni* 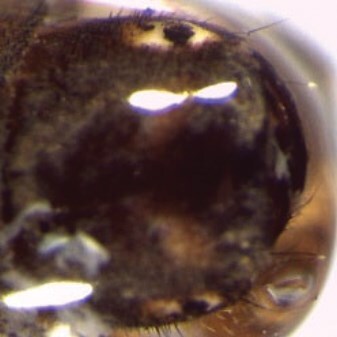	1	22
	2	62		2	273
	3	230		3	441
	4	1		4	2
**Cheumatopsyche oxa** 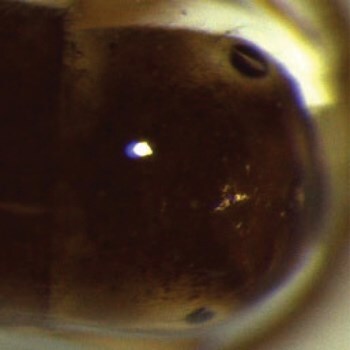	1	2	*Hydropsyche sparna* 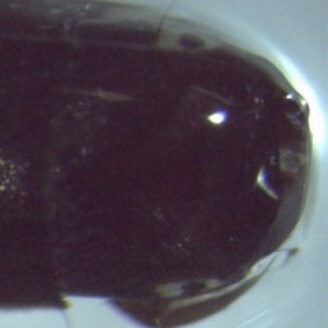	1	1
	2	41		2	49
	3	131		3	281
	4	17		4	4
***Hydropsyche bronta*** 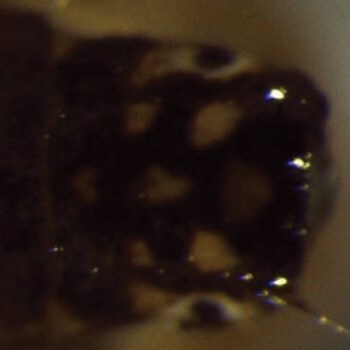	1	3	*Hydropsyche slossonae* 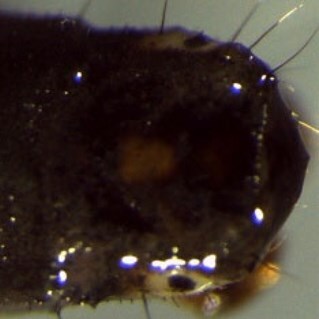	1	7
	2	12		2	76
	3	61		3	58
	4	0		4	4

### Mixed Effect of Agriculture and Cry1Ab Presence on Caddisfly Body Size

**Fig. 2.**
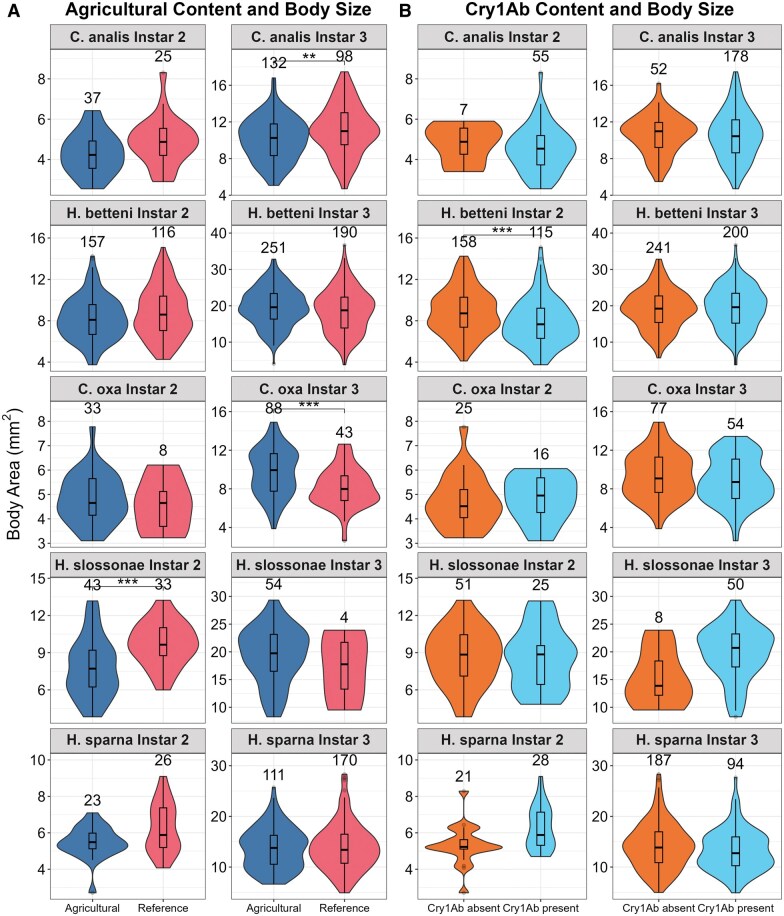
Violin Plots of body area for 5 species of Hydropsychids as a function of (A) land use and (B) Cry1Ab toxin detection in the water column of each stream. Body sizes were compared within instars, and distribution of body area is shown by the width of the violins, with wider sections indicating higher density of individuals. The value above each violin is the sample size per plot. A student’s *t*-test was performed at a significance level of α = 0.05, and the resulting *P*-values are adjusted for multiple comparison using the Bonferroni correction and are illustrated with asterisks where **P < 0.01 and ***P < 0.001.

We first compared body size within frequently observed developmental instars across the 5 most abundant species. This analysis aimed to evaluate potential sublethal effects of agricultural land use and Cry1Ab presence on individual fitness while avoiding confounding from species-level size differences. When comparing by site category (Agricultural vs. Reference, Fig. 2A), 3 species/instar pairs showed significantly different sizes, although the directionality of differences varied across species and instars: For *C. analis*, the third instar was significantly smaller at agricultural sites (*P* = 0.003). For *H. betteni*, the second instar was marginally smaller at agricultural sites (*P* = 0.0376). For C*. oxa*, the third instar was larger at the agricultural sites (*P* < 0.001). For *H. slossonae*, the second instar was smaller at agricultural sites (*P* < 0.001). Finally, for *H. sparna*, the second instar was marginally smaller at agricultural sites (*P* = 0.023).

When comparing between Cry1Ab presence and absence, only the second instar of *H. betteni* showed significantly smaller body size in presence of Cry1Ab (*P* < 0.001). In contrast, both *H. slossonae* instar 3 and *H. sparna* instar 2 were marginally larger with Cry1Ab (*P* = 0.042 and *P* = 0.007, respectively).

In addition to the species-specific comparisons, we also applied a forward-stepping GLMM to jointly evaluate Cry1Ab and multiple ancillary environmental predictors of body size across all individuals and streams within a single modeling framework, while accounting for species identity and instars nested within species (Table 2). Again, we found no effect of crops within the watershed (*P* = 0.247) nor of Cry1Ab presence on insect body size (*P* = 0.222). Instead, we found that 4 other factors better explained differences in body size: impervious surface cover, latitude, stream width, and water temperature (Table 2).

**Table 2.** General linear mixed model estimates on body area

Fixed effects	Estimate	SE	**df**	*t* Value	Pr(>|*t*|)
**(Intercept)**	25.21	8.511	203.8	2.962	0.0034**
**Impervious surface cover**	−5.071	0.9835	1,758	−5.157	2.80e-07***
**Water temperature**	−0.3526	0.05506	1,758	−6.404	1.94e-10***
**Latitude**	−0.306	0.1359	1,756	−2.251	0.0244*
**Stream width**	0.2491	0.05817	1,758	4.282	1.95e-05***
**Crops in watershed**	0.891	0.7698	1,756	1.157	0.2472
**Water pH**	0.496	0.7014	1,759	0.707	0.4795
**Average velocity**	0.2272	0.8346	1,757	0.272	0.7855
**Cry1Ab**	−0.0325	0.02661	1,759	−1.221	0.2221
**Turbidity**	−0.0319	0.02829	1,756	−1.128	0.2596
**Average depth**	0.006258	0.02041	1,760	0.307	0.7592

*p < 0.05; **p < 0.01; ***p < 0.001.

### Developmental Stage within Species

To test whether agriculture and Cry1Ab exposure were associated with delays in larval development, we also compared the average instar stage of individuals within each site. The only species with significant differences in developmental stage between agricultural and Cry1Ab-positive sites was *H. slossonae*. In this species, developmental stage was significantly more advanced in agricultural streams (*P* < 0.001) and in Cry1Ab-positive sites (*P* < 0.001). Developmental stage also increased with water temperature (β = +0.067 instar units per °C, *P* < 0.001). For all other species, no significant differences were detected between agricultural and reference sites, although most showed later developmental stages in warmer streams (Fig. S6, Tables S3 and S4).

### Species Composition Analysis Also Shows no Effect of Cry1Ab

**Fig. 3.**
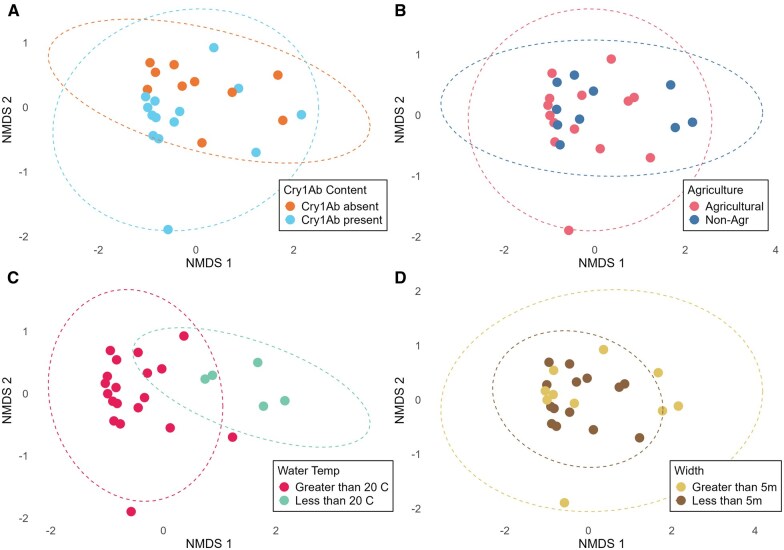
NMDS ordination of species composition across sites based on Bray–Curtis dissimilarity. A) By site category (agricultural development); B) By Cry1Ab presence; C) By site water temperature measured at time of collection, categorized here for visualization purposes; D) By site stream width. Note that variables are categorical for visualization purposes only. In PERMANOVA, they were considered quantitatively.

We also analyzed community composition of Hydropsychidae species to identify differences between agricultural and reference streams, and between those that were Cry1Ab positive vs. no-Cry1Ab (Fig. S8). Across all streams, similar to body size analyses, both Cry1Ab presence (*P* = 0.350) and crop coverage (*P* = 0.726) had small, nonsignificant effects (Fig. 3). The PERMANOVA suggested that water temperature and stream width were the only variables with significant influence on species composition (Table 3).

**Table 3.** Effect of environmental variables on species composition by PERMANOVA across 25 collection sites

	df	SumOfSqs	F.Model	*R*²	Pr(>*F*)
**Latitude**	1	0.034	0.189	0.005	0.974
**Cry1Ab concentration**	1	0.200	1.116	0.028	0.350
**Water pH**	1	0.180	1.004	0.025	0.412
**Water temperature**	1	0.652	3.621	0.091	0.007**
**Turbidity**	1	0.043	0.242	0.006	0.963
**Crops in watershed**	1	0.109	0.610	0.015	0.726
**Impervious surface Cover**	1	0.294	1.636	0.041	0.145
**Stream width**	1	0.684	3.802	0.096	0.009**
**Average stream velocity**	1	0.337	1.873	0.047	0.113
**Average stream depth**	1	0.285	1.584	0.040	0.152
**Residual**	14	2.520		0.354	
**Total**	24	7.118		1	

### Genetic Structure Shows Strong Effect of Geography and No Pattern in Site Category

**Fig. 4.**
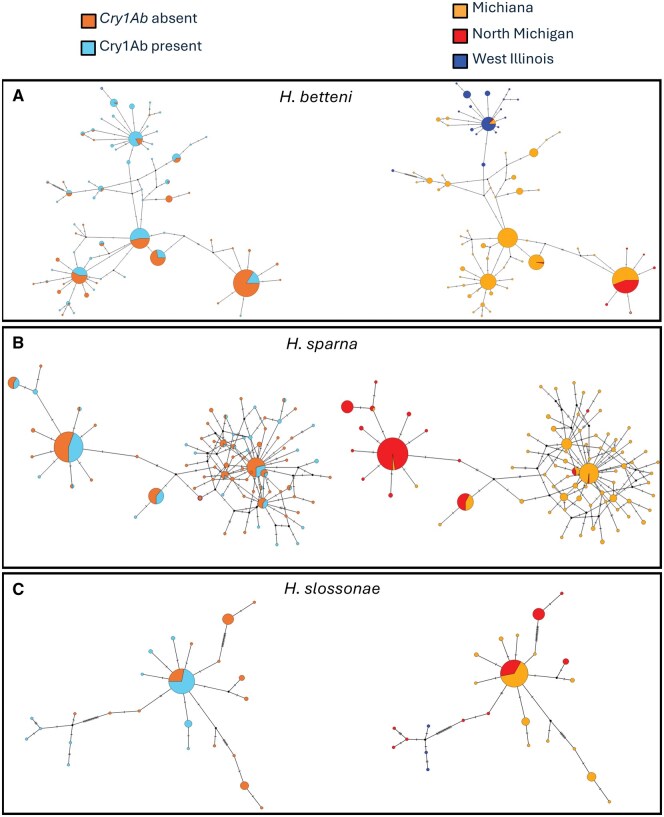
TCS haplotype network of mitochondrial COI gene for 3 representative species. A) *Hydropsyche betteni*, B) *Hydropsyche sparna*, C) *Hydropsyche slossonae*. The haplotype maps on the left are colored to show Cry1Ab (Bt) presence/absence. The haplotype maps on the right are identical but colored based on geography (Fig. S2). Each mark on the line separating nodes indicates 1 SNP, and size of nodes represents haplotype abundance, but size is not constant across panels. Haplotype maps for 4 other species can be found in Fig. S7.

Given that Cry toxins have been in widespread use for nearly 2 decades, it is possible that tolerant or resistant lineages of caddisflies remain in agricultural streams exposed to Cry toxins. If this was the case, we would observe no size or species distribution differences, but we would observe population structure between reference and agricultural sites for affected species. To investigate this possibility, in addition to using genetic barcodes for species identification, we also evaluated population structure at the mitochondrial haplotype level in the most frequently detected species in relation to Cry1Ab exposure or site category (Fig. 4, Fig. S7).

We observed no clear population structuring linked to site category (natural vs. rural) or Cry1Ab presence. Instead, geographic location strongly influenced haplotype patterns in most species. In addition, we found 4 very distinct haplotype groups within individuals annotated as *H. slossonae* (Fig. 4C).

## Discussion

In this study, we investigated the association of the Cry1Ab toxin on natural populations of net-spinning caddisflies (Hydropsychidae) collected across streams in the Midwestern United States. We collected 1,862 individual insects among 12 species, and compared body size, developmental stage, distribution, species composition, and genetic structure (ie haplotype networks) between Cry1Ab positive and negative streams. We documented subtle or minimal effects of Cry1Ab exposure; morphological comparisons revealed mixed evidence for smaller body size and delayed development, while species composition and genetic structure of populations showed no consistent relationship associated with Cry1Ab presence in the water column. Instead, environmental variables such as water temperature, geographic location, and land use were consistently stronger predictors of caddisfly morphology and species composition.

### Limited Impact of Cry1Ab Toxins on Hydropsychid Caddisflies

Among the groups analyzed in this study, early instars of *Hydropsyche betteni* (instar 2) were the only group that exhibited a statistically significant reduction in body size at agricultural sites with measurable Cry1Ab in the water column. In contrast, some instars of of *H. slossonae* and *H. sparna* were marginally but not significantly larger at sites where Cry1Ab was detected. This mixed finding between species is consistent with our *a priori* hypothesis that effects would vary between species, and also consistent with results found in the literature, where the impact of Cry1Ab in other caddisfly groups is also variable (Jensen et al. 2010, Pott et al. 2020), suggesting species-specific effects. Interestingly, all observed size differences were associated with specific instars of these species, and not across multiple instars of a given species. Previous studies have similarly reported that the impact of Cry toxins on insects can vary depending on the developmental instar (Rausell et al. 2000, Hellmich et al. 2001). If Cry or pesticide inputs vary over time following trends in maize growth, then species phenology and developmental stage at the time of peak exposure could influence their susceptibility.

Two alternative hypotheses may explain the marginal increase on body size in other species of caddisflies. First, agricultural sites often receive higher inputs of crop detritus, which may provide an additional source of food for caddisflies, promoting larval growth for some species (Griffiths et al. 2009, Jensen et al. 2010). Nonetheless, we did not find consistently larger larval sizes across agricultural sites, which would be expected if this hypothesis was true. Alternatively, while we did not measure other pesticide levels in the water, caddisflies are known to be sensitive to various chemical pesticides (Liess and Schulz 1996, Yokoyama et al. 2009). If the use of transgenic crops reduces the need for other pesticides, as supported by prior studies (Fitt 2008, Smyth 2020), it is possible that this reduction may have led to a more hospitable aquatic environment in some agricultural sites, indirectly enhancing growth in some species. However, again, we did not measure other pesticides to confirm this association. Overall, these alternatives emphasize that body-size responses in agricultural streams likely reflect multiple interacting influences rather than a single causal factor (Rosi-Marshall et al. 2007).

Furthermore, although minor effects of Cry1Ab were detected in specific comparisons of body size and developmental stage, broader analyses indicate that environmental variables, particularly water temperature and watershed land use, were the dominant drivers of variation in morphology, development, and community composition. This pattern is consistent with Houghton and DeWalt (2023), who identified land use as a major determinant of caddisfly distribution across Midwestern streams. Likewise, species composition in our study was best explained by temperature, stream width, and depth, well-established controls of hydropsychid assemblages due to their niche partitioning and variable mesh sizes (Alstad 1982). In addition, in our analysis of land use, impervious surface cover stood out to us as a potential influencer of caddisfly health. While not intrinsically linked to our categorization of reference or rural, we looked at this type of land use due to increased stream pollution associated with urbanization (Walsh et al. 2005). Other studies on other aquatic macroinvertebrates indicate a negative impact on species richness due to increased impervious surface cover (Fogaça et al. 2013, Westman and Martin 2025). Our study was somewhat consistent with these data as our linear model indicated a negative estimate on body size due to the effort of impervious surface cover (Table 2). However, species composition did not seem affected (Table 3). Nonetheless, because caddisfly diversity is often used as a bioindicator of freshwater ecosystem health (McCahon et al. 1989, Wiggins 1996, Hamid and Rawi 2017), any future changes in their diversity, even if not directly linked to Cry toxins, may still signal shifts in water quality driven by broader land use and environmental change. Our results, however, seem to suggest that Hydropsychidae may not be a good bioindicator in agricultural streams, which has a precedent in caddisflies in the *Smicridea* genus found in the Colorado River Basin (Cavallaro 2026).

Finally, previous studies have reported delays in caddisfly and other insect development following Cry1Ab exposure (Jensen et al. 2010, Pott et al. 2020). In our data, we observe minor effects of Cry1Ab on caddisfly developmental stage. While *H. slossonae* showed developmental shifts at Cry1Ab-positive sites, this nominal species likely encompasses multiple cryptic species (Fig. 4C, see below), and these patterns were weak relative to the strong and consistent influence of water temperature (Table 3). This is supported by previous work documenting the central role of temperature in aquatic insect growth (eg Suzuki et al. 2024). While temperature had a significant effect as a variable, it is important to recognize temperature was correlated with tree cover and significantly correlated with stream depth, which could be factors as well (Fig. S12). Nonetheless, our study design is not appropriate to isolate these variables.

### Limitations

While we found little to no evidence of Cry1Ab exposure impacting larval Hydropsychidae caddisflies, these findings are shaped by inherent methodological constraints. The observational nature of our study limits our ability to attribute differences in caddisfly morphology or development solely to exposure of a single Cry variant. Additionally, only larval caddisflies were examined in this study, and we do not know if the adult phases were affected morphologically or developmentally. Additional study on emergence phenology in these streams may provide information we were not able to obtain given our methods. Agricultural streams experience multiple overlapping stressors, including elevated nutrient loads, sedimentation, habitat alteration, and exposure to a variety of agrochemicals (Lange et al. 2014, Piggott et al. 2015, Cornejo et al. 2019). We were unable to isolate these confounding influences, nor did we quantify competitive interactions or mortality—important fitness-related metrics often assessed in controlled experiments (eg Rosi-Marshall et al. 2007).

In addition, a key limitation in assessing the ecological impacts of Cry toxins is the lack of publicly available data on the specific transgenic seed varieties planted near sampling sites, as US producers are not required to report when or where particular seed types are deployed (Marvier et al. 2008). Consequently, the identity and abundance of Cry variants present in a watershed over time remain uncertain, preventing precise inference of exposure histories for nontarget organisms. Our analyses focused on Cry1Ab, but other Cry variants are likely present at similar or greater concentrations. Although our assay cross-detects related proteins (Cry1Ac, Cry1A.105, Cry2Ab2; Brandão-Dias et al. 2021), these were not quantified separately, and the total Cry burden may therefore be underestimated.

### Haplotype Structure and Cryptic Diversity

While we detected no genetic structure associated with Cry1Ab exposure or site category, we observed substantial mitochondrial genetic variability and clear regional clustering within most species. Such population structuring has been repeatedly documented in aquatic insects and is typically attributed to limited dispersal ability, localized recruitment, and the strong influence of watershed boundaries on gene flow (Lehrian et al. 2010, Sproul et al. 2014, Metcalfe et al. 2020). These patterns suggest that regional environmental gradients and historical connectivity, rather than recent exposure to Cry toxins, likely explain the genetic differentiation observed here. Nonetheless, our study area lies within a heavily farmed landscape, where habitat alteration, nutrient runoff, and pesticide use have been linked to reduced genetic diversity in aquatic invertebrates (Crossley et al. 2023). Thus, it is possible that the high genetic diversity observed here is a sign of no recent bottlenecks in these populations, a potential good sign for their status.

A notable pattern emerged in *Hydropsyche slossonae*, where 4 distinct haplotype groups were observed. This pattern could indicate cryptic species, but an alternative explanation is misannotation of sequences in public databases like GenBank, where multiple described species may have been incorrectly submitted under the name *H. slossonae*. Resolving whether these groups represent true species-level divergence or database artifacts will require additional markers and more detailed taxonomic validation. Regardless, the presence of multiple distinct haplotype groups within nominal *H. slossonae* highlights the potential for unrecognized diversity in caddisflies in the Midwestern United States.

In addition, we found individuals genetically assigned to *H. alhedra* in one of our sites in Western Illinois. Based on the Nearctic checklist for trichopterans (Rasmussen and Morse 2023), *H. alhedra* is not currently known to Illinois (Hughlett Branch, Table S2), so that is likely a new report for the state. It is possible this detection encompasses a range expansion or population recovery from extirpations prior to extensive monitoring, but studies are needed to evaluate if this represents an established population.

## Conclusion

In conclusion, our results indicate that Cry1Ab exposure in agricultural streams is not greatly associated with morphological, developmental, or genetic variation in larval Hydropsychid caddisflies under natural field conditions. Environmental variables such as temperature, stream form, and land use were consistently stronger predictors of body size, developmental stage, and community composition. These findings suggest that natural environmental gradients, rather than Cry protein exposure, structure caddisfly populations across Midwestern streams, though small or context-dependent effects cannot be ruled out. Continued field monitoring that integrates environmental data, Cry protein concentrations, and population-genetic information will be essential to detect potential cumulative or context-dependent effects. The strong regional genetic structure and unrecognized diversity documented here highlight the importance of incorporating molecular approaches into biomonitoring and ecological risk assessment for aquatic invertebrates.

## Supplementary Material

nvag025_Supplementary_Data
