# Supplementary material for: Assessing morphological, developmental, and genetic responses of Hydropsychid caddisflies to Cry1Ab exposure
Source: Environ Entomol. 2026 Apr 7;55(2):nvag025. doi: 10.1093/ee/nvag025 (PMC13064931; doi:10.1093/ee/nvag025)
Supplement: nvag025_Supplementary_Data [file nvag025_supplementary_data.zip › Supplement_submission_Review1.pdf]

Supplementary Information for

Assessing Morphological, Developmental, and Genetic Responses of Hydropsychid Caddisflies to Cry1Ab Exposure

Ethan P Bull[1], Scott P. Egan[1]*, Jennifer L Tank[2], Elise D Snyder[2,3], Yufei Qi[1], Amanda Potts[1], Maryam Rajabi Faghihi[1], and Pedro FP Brandão-Dias[1,4]*

1: Department of Biosciences, Rice University, Houston, Texas, USA

2: Department of Biological Sciences, University of Notre Dame, Notre Dame, Indiana, USA

3: Illinois Natural History Survey, Champaign, Illinois, USA

4: School of Marine and Environmental Affairs, University of Washington, Seattle, Washington, USA

*Corresponding authors. E-mails: spe1@rice.edu, pedro_bdfp@hotmail.com

Table of Contents

Figure S1 2
Figure S2 2
Figure S3 3
Figure S4 3
Figure S5 4
Figure S6 5
Figure S7 6
Figure S8 7
Figure S9 8
Figure S10 9
Figure S11 10
Figure S12 11
Table S1 12
Table S2 13
Table S3 14
Table S4 15
Table S5 16

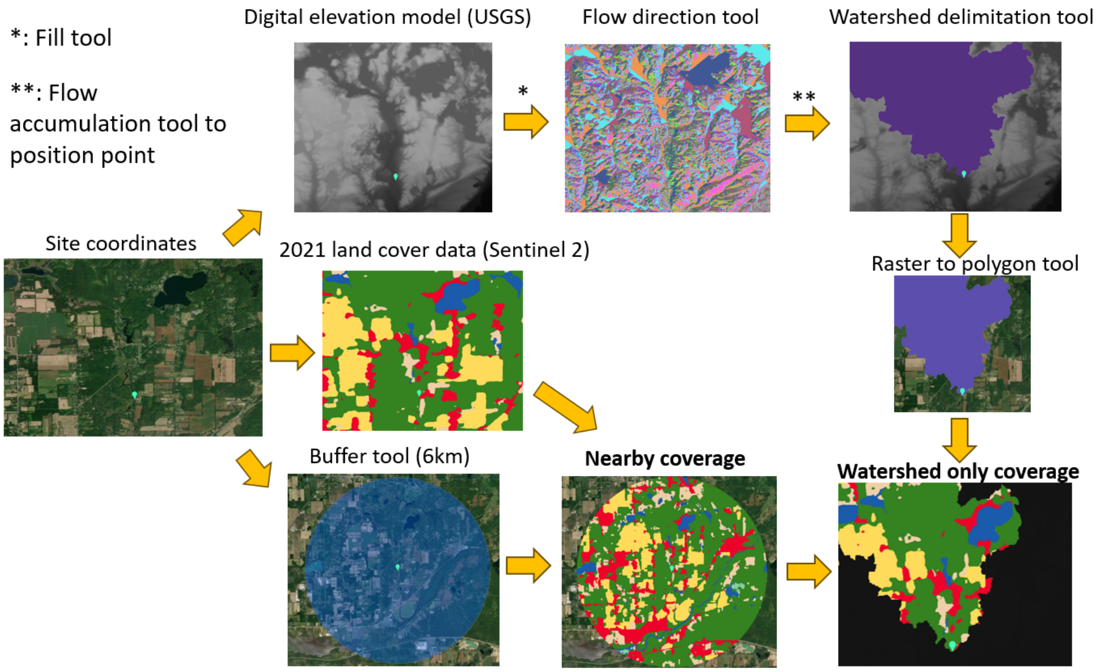

Figure S1: ArcGIS method workflow for an example site.

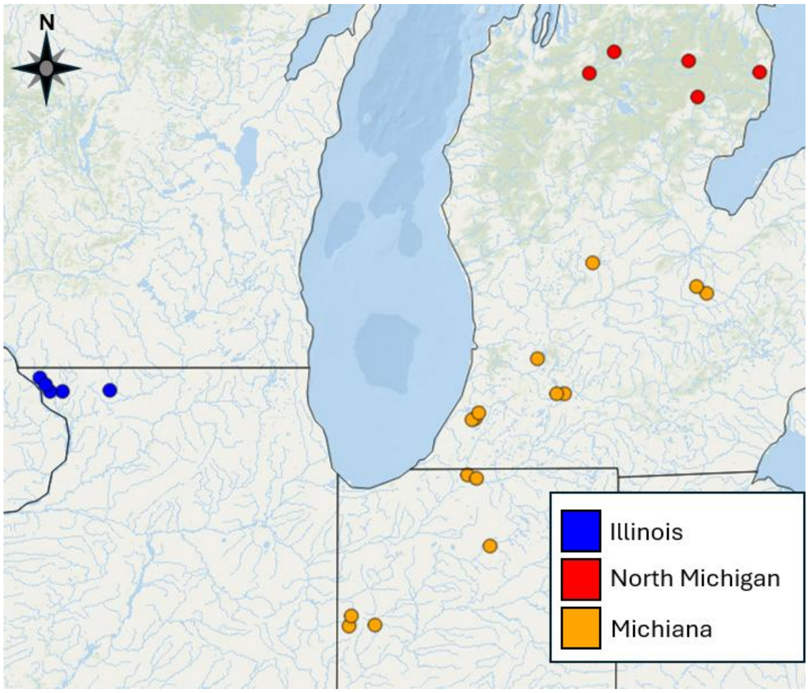

Figure S2: Map of sampled sites colored by region assignments. Same colors and regions used in the haplotype maps

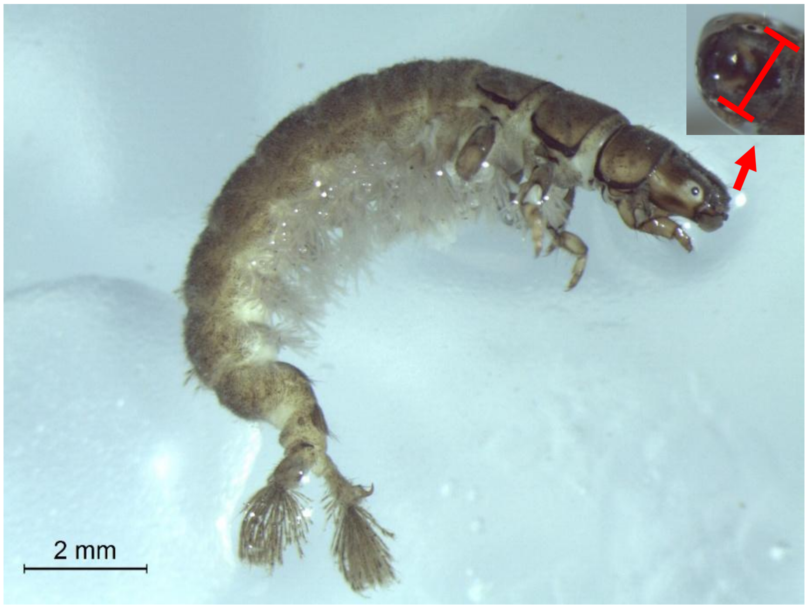

Figure S3. Morphological Measurements of Caddisflies. Each caddisfly was laid on its side and 2 measurements were taken using a Leica M125 Stereoscope using Leica Application Suite. Head capsule width ImageJ contrast area (S2) of the caddisfly was measured to the nearest .001 mm^2. Picture here is individual #100, a *Hydropsyche betteni* Collected from PTC site

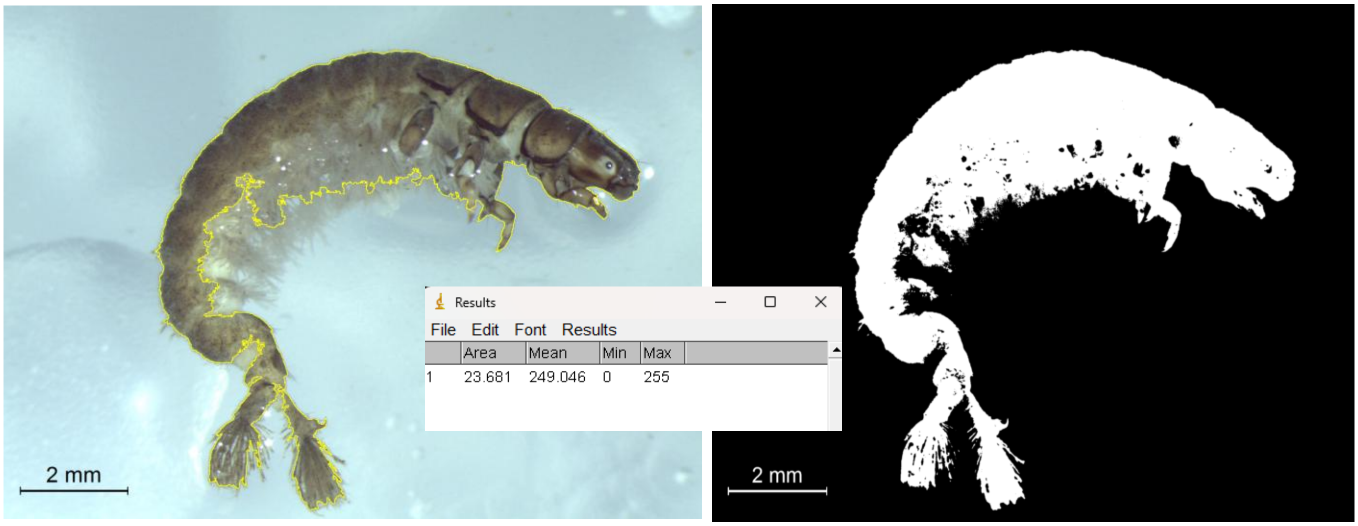

Figure S4. ImageJ analysis of caddisfly. Using the scale from the stereoscope and ImageJ wand tool for contrast selection, a side area of the caddisfly was generated to the nearest .001 mm^2.

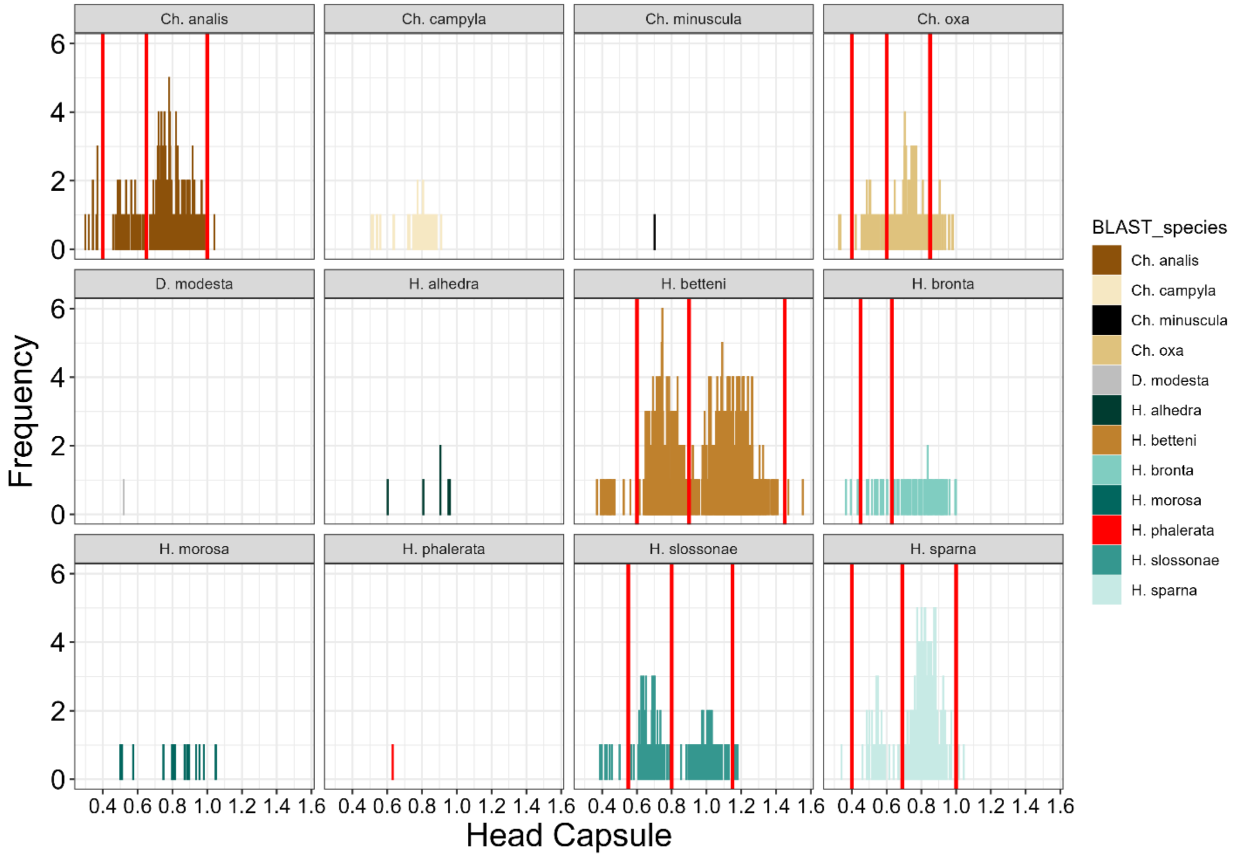

Figure S5. Frequency Histogram of caddisfly head capsule width (mm^2). Instars are determined based on natural splits in the data (table 2).

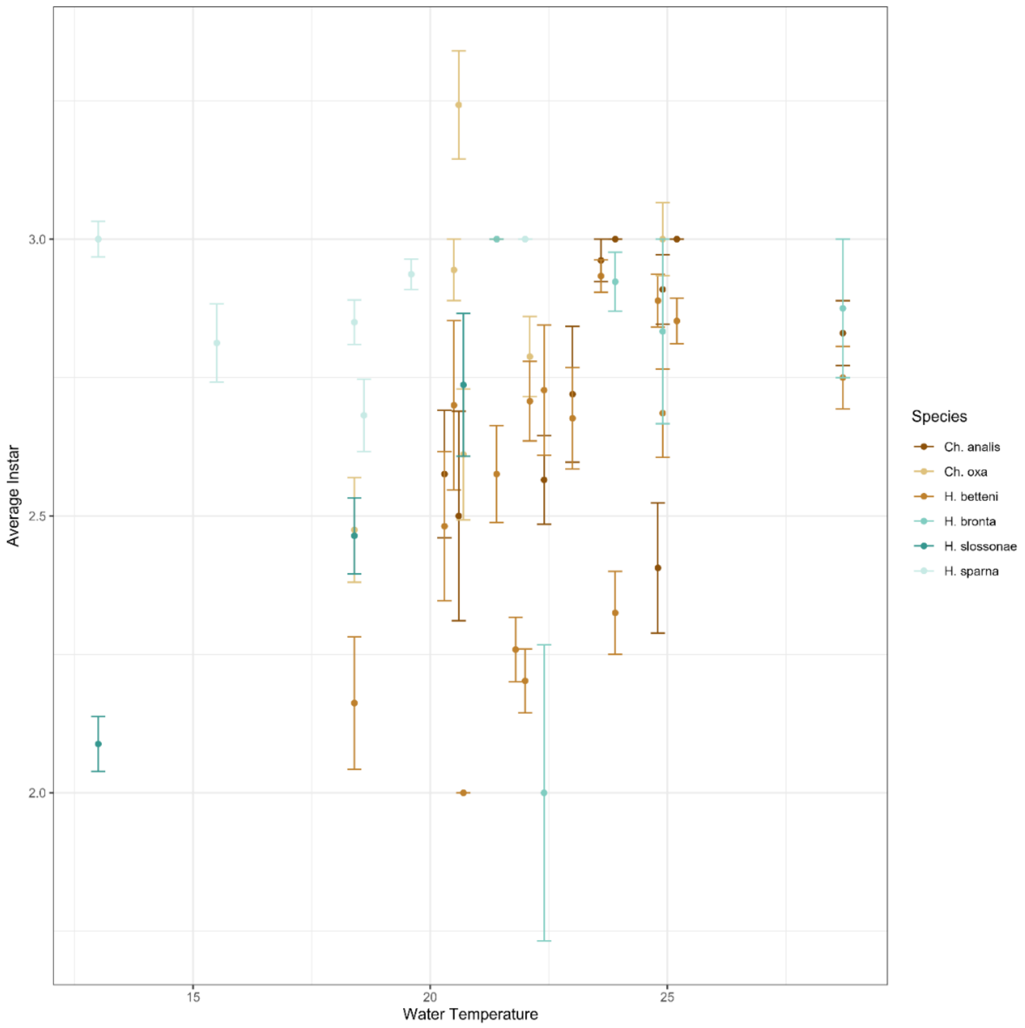

Figure S6. Instar Score colored by species, arranged by water temperature. Includes 95% confidence

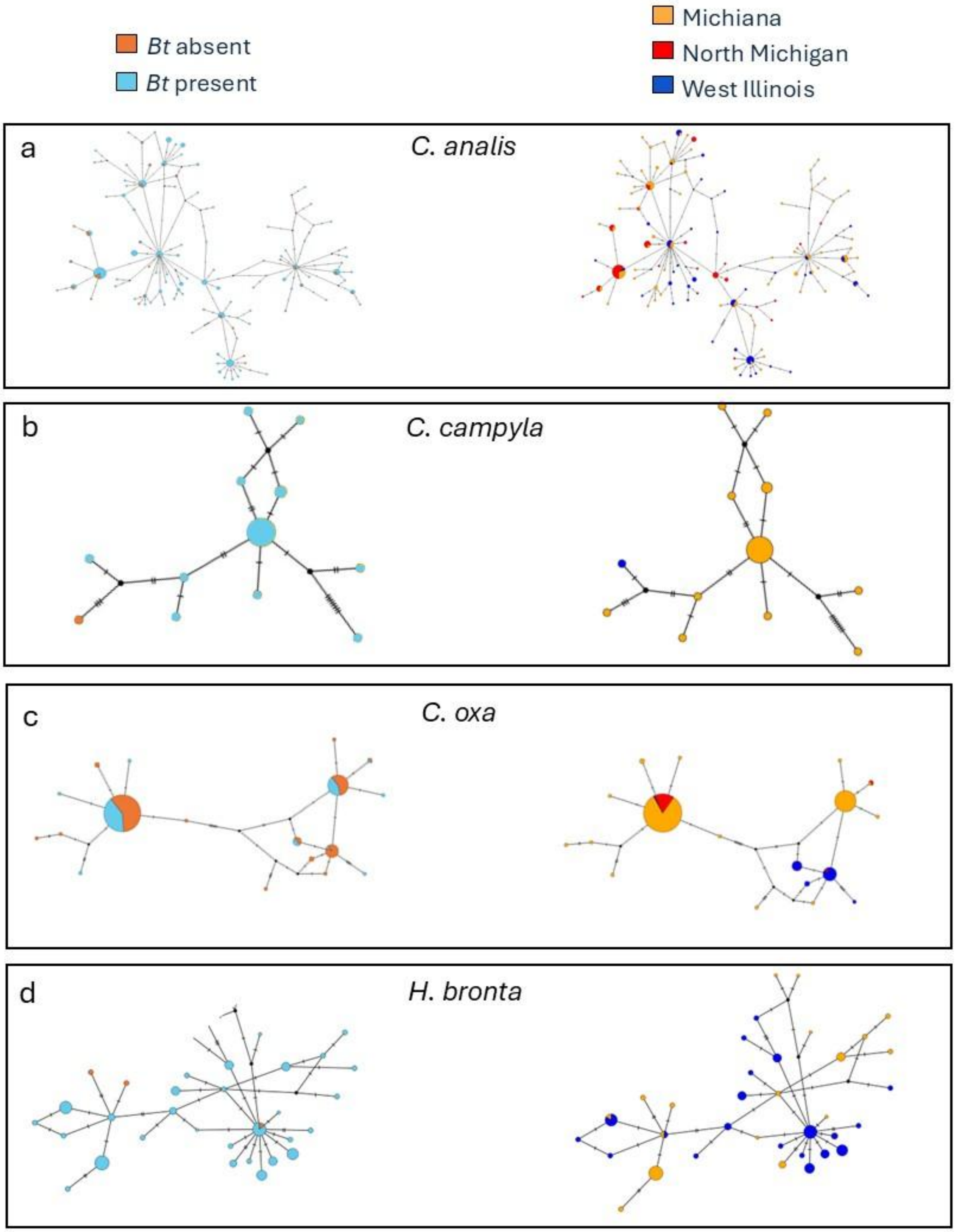

Figure S7. TCS Haplotype Network of four additional species. (a) Cheumatopsyche analis; (b) Cheumatopsyche campyla; (c) Cheumatopsyche oxa; (d) Hydropsyche bronta. Maps on the left are colored by Bt- Cry Content, whereas maps on the right are colored by Geography. Haplotype maps for other 3 species can be found in the main text.

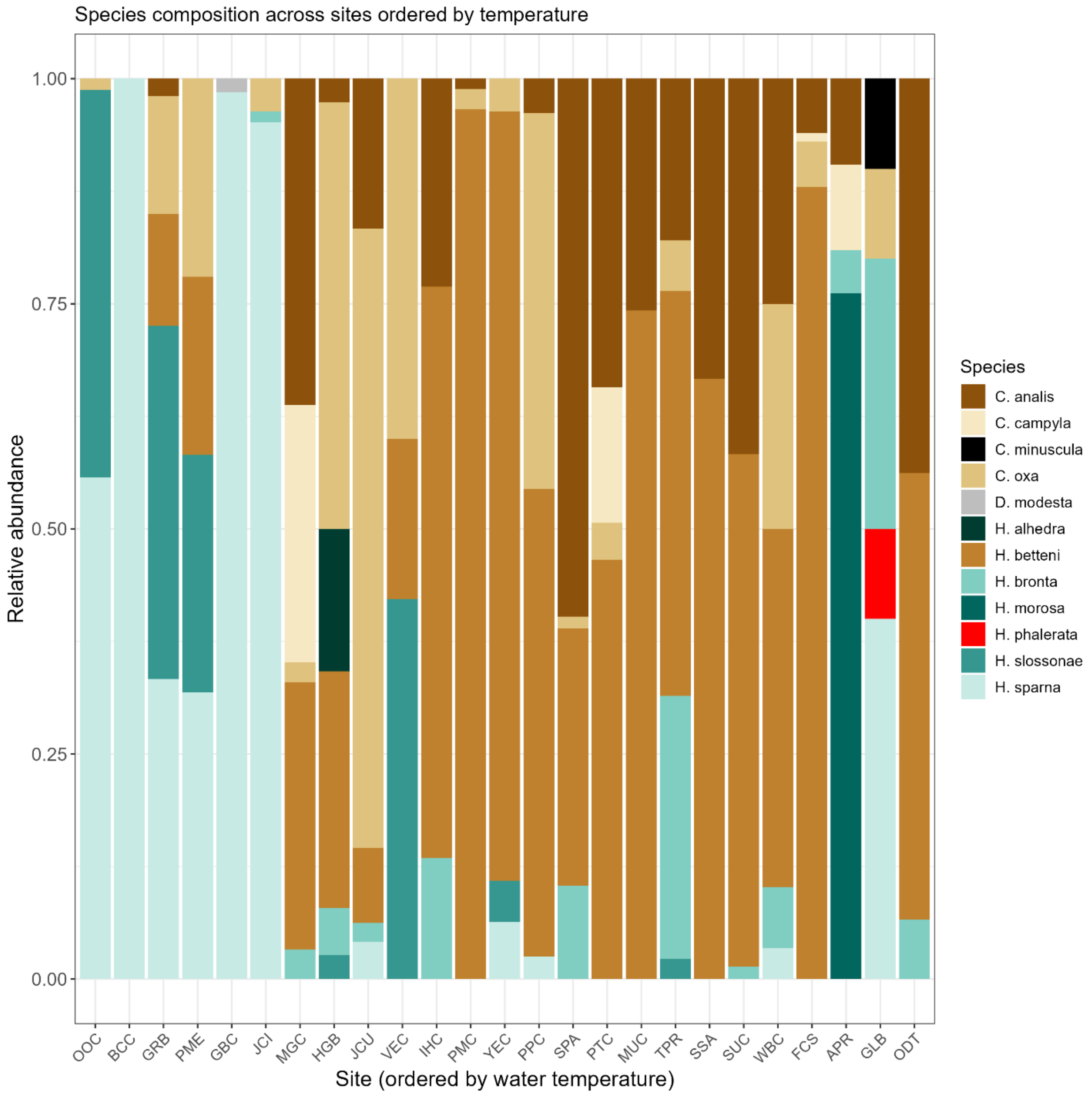

Figure S8. Species Compositions found at different Water Temperatures. Note that the temperature measured instantaneously at the time of sampling may not necessarily represent the stream's thermal regime.

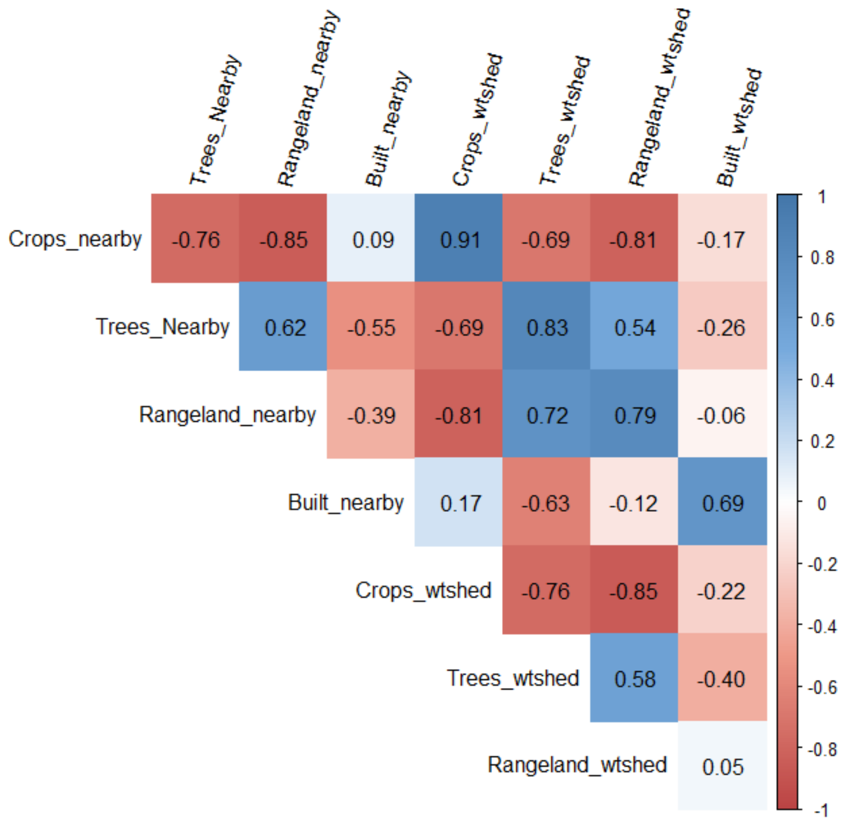

Figure S9. Correlational plot of ArcGIS-measured variables, used to select which variables that were not strongly correlated with crops in watershed. Building in the watershed was easy to justify as it was not strongly correlated with crops in the watershed.

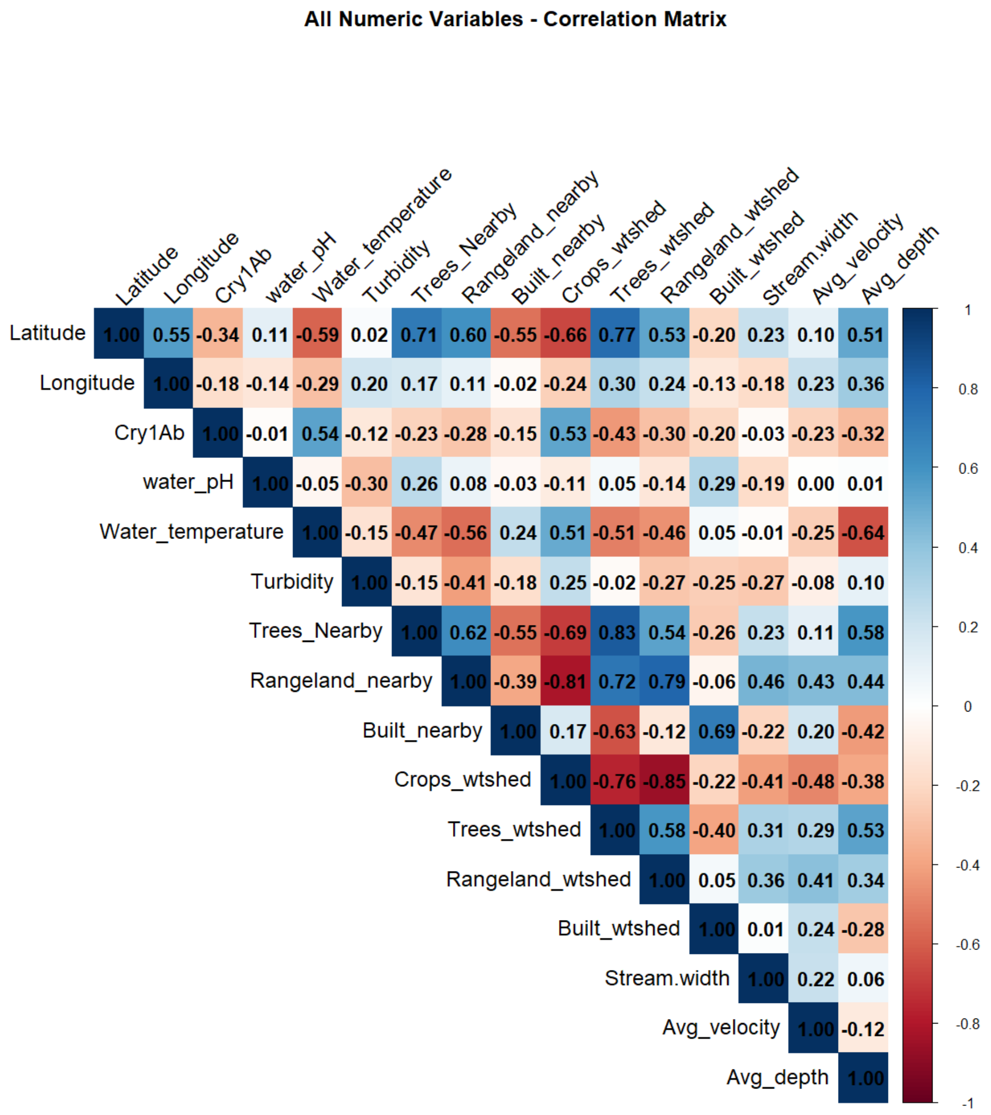

Figure S10. Correlational plot of all variables. Exclusion criteria for fit into our GLMM were first important variables for the hypothesis (crops in watershed), then other variables with |r| < 0.7.

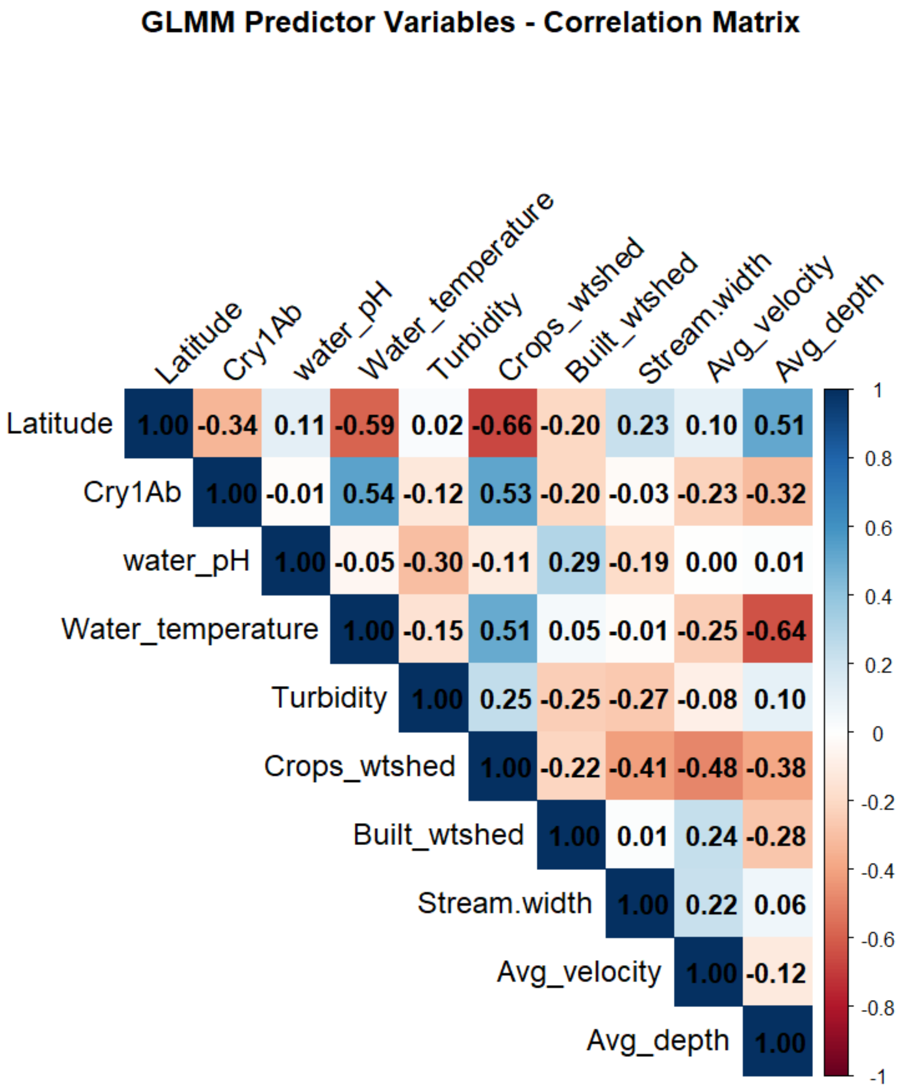

Figure S11. Correlational plot of GLMM variables.

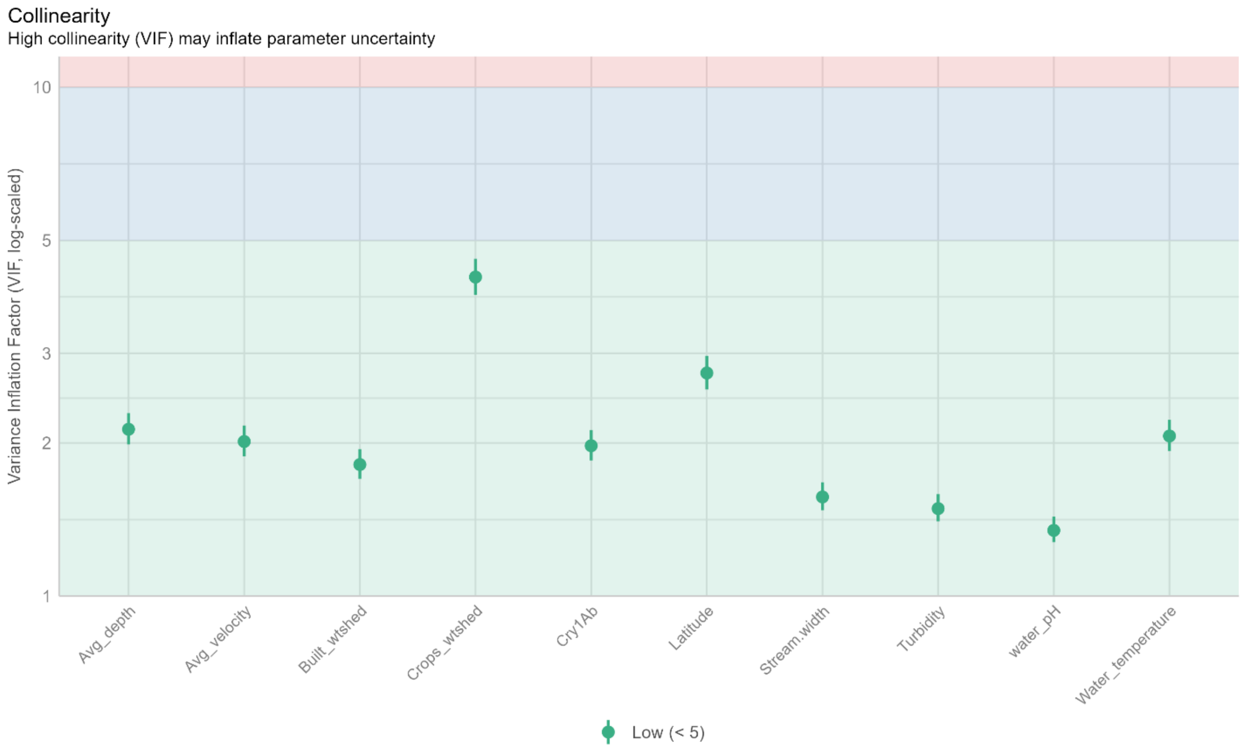

Figure S12. Collinearity plot of GLMM variables (VIF)

Table S1. Delimitation of instars for analysis. Instars were determined based on head capsule width frequency plot groupings of each species.

Ch. analis		*H. betteni*		*Ch. oxa*	
**Instar**	**Head Capsule Width Range (mm)**	**Instar**	**Head Capsule Width Range (mm)**	**Instar**	**Head Capsule Width Range (mm)**
1	<0.4	1	<0.6	1	<0.4
2	0.4-0.65	2	0.6-0.9	2	0.4-0.6
3	0.65-1	3	0.9-1.45	3	0.6-0.85
4	>1	4	>1.45	4	>0.85

H. sparna		*H. bronta*		*H. slossonae*	
**Instar**	**Head Capsule Width Range (mm)**	**Instar**	**Head Capsule Width Range (mm)**	**Instar**	**Head Capsule Width Range (mm)**
1	<0.4	1	<0.45	1	<0.55
2	0.4-0.69	2	0.45-0.63	2	0.55-0.8
3	0.69-1	3	>0.63	3	0.8-1.15
4	>1			4	>1.15

Table S2. Location data and agricultural delimitation

Site Name	State	Site	Latitude	Longitude	Agriculture
**Juday Creek Isaac Walton L**	IN	JCI	41.7266	-86.2642	Reference
**Juday Creek Upstream**	IN	JCU	41.70269	-86.1788	Agricultural
**Apple River**	IL	APR	42.3354	-90.2071	Agricultural
**Hughlett Branch**	IL	HGB	42.4282	-90.426	Reference
**Irish Hollow Creek**	IL	IHC	42.33055	-90.329	Reference
**Smallpox Creek A**	IL	SPA	42.3818	-90.3734	Reference
**Tributary of Pecatonica River**	IL	TPR	42.3387	-89.749	Agricultural
**Fort Custer**	MI	FCS	42.31721	-85.3171	Reference
**Gull Creek**	MI	GLB	42.32139	-85.4014	Agricultural
**Misteguay Creek**	MI	MGC	43.03484	-83.9332	Agricultural
**Porter Creek**	MI	PTC	43.08733	-84.0336	Reference
**Mud Creek 1F**	IN	MUC	40.62594	-87.4176	Agricultural
**Owens Ditch 1D**	IN	ODT	40.62868	-87.1681	Agricultural
**Sugar Creek 1C**	IN	SUC	40.69795	-87.4008	Agricultural
**Shatto_1**	IN	SSA	41.21371	-86.0453	Agricultural
**Greegs Brook**	MI	GRB	42.56862	-85.5824	Agricultural
**West Branch Fish Creek**	MI	WBC	43.2497	-85.0418	Reference
**Big Cannon Creek**	MI	BCC	44.58324	-85.0734	Agricultural
**Cherry Creek**	MI	YEC	44.66842	-84.11	Reference
**Gamble Creek**	MI	GBC	44.42143	-84.0285	Reference
**Goose Creek**	MI	OOC	44.73355	-84.8379	Reference
**Van Etten Creek**	MI	VEC	44.59074	-83.4146	Reference
**Pawpaw_M4**	MI	PMC	42.14	-86.1856	Agricultural
**Pawpaw_M6**	MI	PME	42.12929	-86.2207	Agricultural
**Pawpaw_P3**	MI	PPC	42.17861	-86.1575	Agricultural

Table S3. Mann-Whitney U Test and OLS for Caddisfly Developmental Factors.

	**Mann-Whitney U Agriculture**		**Mann-Whitney U Bt**		**OLS Regression**	
	W_Agriculture	P_Agriculture	W_BtContent	P_BtContent	Slope_WaterTemp	P_WaterTemp
**Ch. analis**	9850	0.992	6186	0.062	0.021	0.067
**H. betteni**	66113	0.819	64794	0.366	0.073	<0.001
**Ch. oxa**	3322	0.082	4757.5	0.118	0.082	<0.001
**H. slossonae**	2917	<0.001	1300	<0.001	0.067	<0.001
**H. sparna**	13150	0.492	14897.5	<0.001	-0.004	0.635
**H. bronta**	586	0.199	154	0.304	0.053	0.049

Table S4. Mean Instar score and number reporting

	Instar Score	Agricultural	Reference	Bt Absent	Bt Present
**Ch. analis**	n	176	112	45	243
	Mean	2.73	2.73	2.87	2.70
**H. betteni**	n	422	316	415	323
	Mean	2.57	2.58	2.56	2.59
**Ch. oxa**	n	134	57	119	72
	Mean	2.81	2.96	2.91	2.76
**H. slossonae**	n	105	40	64	81
	Mean	2.52	2.10	2.09	2.65
**H. sparna**	n	136	199	211	124
	Mean	2.85	2.87	2.91	2.77
**H. bronta**	n	43	24	4	63
	Mean	2.81	2.62	3.00	2.73

Table S5. Variance Inflation Factor of variables used in the GLMM

Variable	VIF
Crops in watershed	4.24
Latitude	2.74
Average stream depth	2.13
Water temperature	2.06
Average stream velocity	2.01
Cry1Ab concentration	1.98
Impervious Surface Cover	1.81
Stream width	1.57
Turbidity	1.49
Water pH	1.35
